# Tulip-shaped vs. classic gel supraglottic airway device on hypopharyngeal secretion pH: a randomised clinical trial

**DOI:** 10.1515/med-2026-1535

**Published:** 2026-11-02

**Authors:** Yonghui Wang, Kaili Qu, Xiaoshuang Tuo, Fahan Wang, Yingying Wang, Yunfei Qi, Zhihong Lu

**Affiliations:** Department of Anaesthesiology and Perioperative Medicine, Key Laboratory of Anaesthesiology of Ministry of Education, Shaanxi Provincial Key Laboratory of Traditional Chinese Medicine, Xijing Hospital, Fourth Military Medical University, Xi’an, Shaanxi, China; Department of Anaesthesiology, 949 Hospital, Altay, Xinjiang, China

**Keywords:** supraglottic airway devices, regurgitation, leak pressure, general anaesthesia, GMA-Tulip, i-gel

## Abstract

**Objectives:**

Risk of regurgitation is an inherent limitation of supraglottic airway devices (SADs). This study aims to determine whether tulip-shaped gel SAD could affect hypopharyngeal secretion pH which may reflect risk of regurgitation.

**Methods:**

This single-blind, randomised clinical trial was conducted from August 2024 to January 2025 at two hospitals in China. Patients aged over 18 years and scheduled for elective surgery were randomised to receive either tulip-shaped (GMA-Tulip) or classic gel SAD (i-gel). The primary outcome was the pH value of secretion at the tip of the SAD upon removal.

**Results:**

A total of 72 patients were randomly assigned. The mITT analysis showed that the primary outcome was comparable between the Tulip and the Classic group (mean [SD]: 6.8 [0.7] vs. 6.9 [0.7], MD [95 % CI]: −0.04 [−0.37, 0.30]; p=0.830). No episode of gastrooesophageal reflux was observed in either group. Secondary observations suggested potentially shorter time to effective ventilation (23.0 [10.7] sec vs. 49.2 [64.8] sec; p=0.022) and fewer insertion attempts (1 [0] vs. 1 [0.75], p=0.026) with the Tulip device, but these findings are exploratory.

**Conclusions:**

Tulip-shaped gel SAD did not result in a significant difference in the pH of secretions at the device tip compared to i-gel. Clinical trial registration: ClinicalTrial.gov (NCT06527352), https://clinicaltrials.gov.

## Introduction

The use of supraglottic airway devices (SADs) represents a significant advancement in airway management and may provide a superior alternative to airway management for short, superficial surgeries and for patients unable to tolerate tracheal intubation [1]. Compared to endotracheal tubes, the use of SADs helps improve haemodynamic and respiratory stability during induction and emergence from anaesthesia and may reduce anaesthetic requirements. However, SADs also have inherent limitations, with one major risk being the inability to provide a complete seal of the airway, thus failing to prevent regurgitation and aspiration [1]. Second-generation SADs are designed with a channel for gastric tube placement to decompress gastric contents and reduce the risk of regurgitation, although this risk remains [2], 3]. There is a wide variety of SADs, and in recent years, SADs with non-inflatable gel cuffs have developed rapidly. Their shape closely conforms to the anatomical structure of the pharyngeal tissue, achieving a good seal and satisfactory positive-pressure ventilation [1].

Given the need for a close match between SAD shape and the laryngeal inlet, numerous innovative designs have emerged in recent years. Among these, the Tulip-like SAD (GMA-Tulip) features a tulip-shaped tip (Figure 1), which theoretically mirrors the glottic anatomy on the glottis and allows for better conformity to the bilateral piriform fossa, potentially reducing gastrooesophageal reflux and regurgitation. Furthermore, the design permits passage of two gastric tubes, which may enhance gastric content drainage [4]. However, there are currently no reports on the effect of tulip-shaped SAD on reflux or regurgitation. The pH value of hypopharyngeal secretion may reflect the risk of regurgitation. Therefore, the objective of this study was to compare pH value of hypopharyngeal secretion at the end of the surgery between the Tulip-shaped and the classic gel SAD, aiming to inform airway management in patients under general anaesthesia.

**Figure 1:**
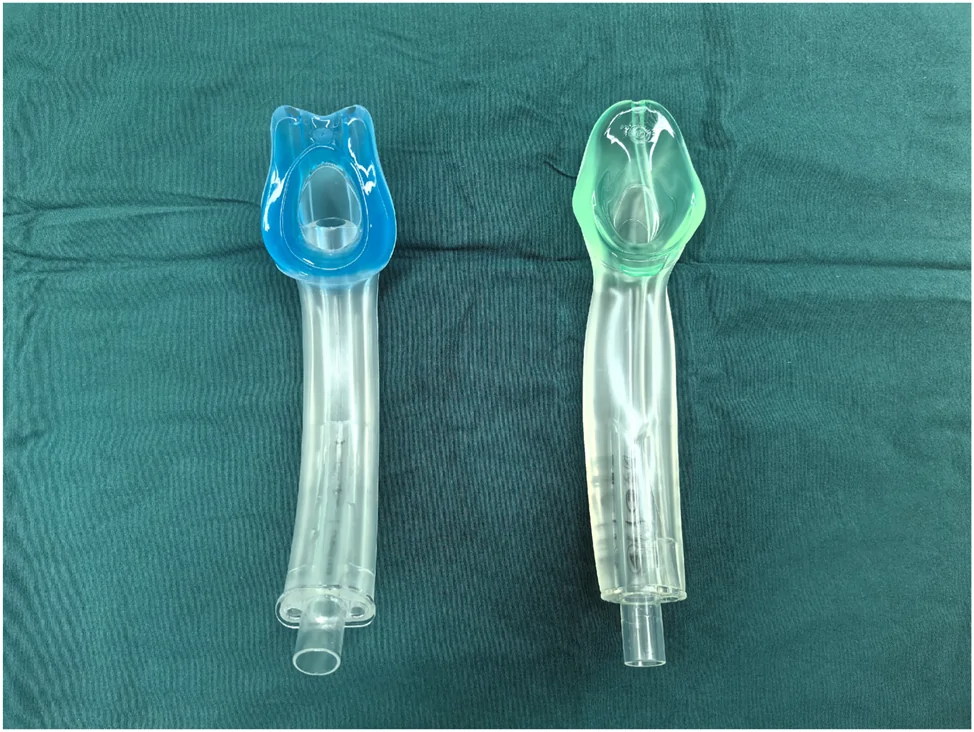
Supraglottic airway devices used in the study.

## Materials and methods

### Study overview

The study was conducted at two regional tertiary hospitals in China: Xijing Hospital and the 949th Hospital. This was a dual-centre, randomised, patient-blind, parallel-controlled trial. The study was approved by the institutional ethics committee (KY20242262-C-1) and registered on clinicaltrials.gov (NCT 06527352). All participants provided written informed consent. The study adhered to the Helsinki Declaration, and the report followed the CONSORT guidelines.

### Patients

Patients aged ≥18 years scheduled for surgery under general anaesthesia using a laryngeal mask airway were screened. Exclusion criteria included American Society of Anaesthesiologists (ASA) physical status >III; morbid obesity (BMI >40 kg m^−2^); high risk of reflux or aspiration (e.g., symptomatic gastrooesophageal reflux or hiatal hernia); anticipated difficult airway; and airway diseases (sore throat or respiratory infection).

### Randomisation and blinding

Patients were randomly assigned in a 1:1 ratio to the Tulip group or the classic group using a computer-generated random sequence (PASS 15.0 software). Randomisation information was sealed in opaque, sequentially numbered envelopes and opened only after the participant entered the operating room. Participants were blinded to group allocation. While the attending anaesthesiologist inserting the SAD could not be blinded to device type due to visible differences, the outcome assessor for pH measurement and postoperative follow-up was independent and blinded to group allocation. The pH paper was read only after SAD removal, without the assessor observing the device.

### Study intervention

Participants were randomly assigned to the Tulip group or the classic group, receiving the Tulip shaped SAD (GMA-Tulip, Tianjin Medan Medical Corp, China) or the classic gel SAD (i-gel, Intersurgical Ltd, Berkshire United Kingdom) (Figure 1), respectively. At each centre, SAD placement was performed by the same designated researcher.

On arrival at the operating room, standard monitoring was established, including electrocardiography, non-invasive or invasive blood pressure, and pulse oximetry. After 3 min of preoxygenation via face mask, anaesthesia was induced with midazolam (0.03 mg/kg), propofol (target-controlled infusion, plasma target concentration of 3 μg/mL), sufentanil (0.3 μg/kg), and dexamethasone 0.1 mg/kg. Once the patient lost consciousness (Modified Observer’s Assessment of Alertness/Sedation [MOAA/S] score=0, defined as no response to trapezius squeeze), rocuronium 0.6 mg/kg was administered intravenously. The GMA-Tulip or i-gel was inserted according to the assigned group. After placement, the SAD was secured with tape and 14-Fr gastric tube pre-lubricated with liquid paraffin was inserted through the gastric drainage port. Correct placement of gastric tube was confirmed by aspiration of gastric contents. Gastric decompression was performed, and the volume of aspirated gastric fluid was recorded. Anaesthesia was maintained with sevoflurane and remifentanil. For laparoscopic procedures, pneumoperitoneum pressure was maintained at 12–14 mmHg according to institutional protocol. Ventilation was adjusted to maintain ETCO_2_ between 35 and 45 mmHg, with peak airway pressures consistently below 25 cmH_2_O. Parecoxib sodium and palonosetron were given 30 min before the end of surgery to reduce postoperative pain and nausea/vomiting. At the end of surgery, the laryngeal mask was removed when the patient opened their eyes to command and could follow instructions. Neuromuscular blockade was monitored using train-of-four stimulation throughout surgery in all patients and sugammadex (2 mg/kg) was administered for reversal as needed.

Upon removal, secretions at the tip of the SAD were collected immediately (<1 min) using MQuant^®^ pH indicator strips (Merck KGaA, Darmstadt, Germany; range 0–14, resolution 0.5 pH units). The paper was applied to the lowest point of the SAD bowl for approximately 5 s until color stabilization, and the reading was recorded by comparison with the manufacturer’s reference color chart. Measurements were performed once per patient due to the limited volume of secretion available.

### Outcome measure

The primary outcome was the pH value of secretions at the tip of the SAD upon removal, measured using pH indicator strips.

Secondary outcomes included: pH gradient between the tip and the back of the SAD upon removal; intraoperative incidence of gastrooesophageal reflux, defined as a hypopharyngeal pH <4 upon removal [5]); oropharyngeal leak pressure (OLP) (the steady-state airway pressure with the adjustable pressure limiting valve setting to 40 cmH_2_O at a fixed gas flow of 3 L/min [6]); time and number of attempts for laryngeal mask insertion (from the operator picking up the laryngeal mask to successful placement and connection to the anaesthesia machine); time to establish effective ventilation (from SAD insertion into the mouth to the first appearance of the ETCO_2_ waveform); epiglottic downfolding observed with fibrescope; fibroscopic grading of SAD position (0=optimal, 1=adequate ventilation but lateral deviation, 2=ventilation compromised); intraoperative adjustments of SAD position (e.g., head and neck repositioning, adjustment of insertion depth, jaw thrust); difficulty of gastric tube insertion (0=no difficulty, 1=resistance but resolved quickly, 2=failure); incidence of blood staining on the SAD; anaesthesiologist satisfaction with the SAD management (0–10, where 0=very dissatisfied, 10=very satisfied); incidence of sore throat, hoarseness, dysphonia and postoperative nausea and vomiting (PONV) in post-anaesthesia care unit (PACU) and at 24 h after surgery.

### Statistical analysis

Based on previous report [7], the mean hypopharyngeal pH at the end of surgery would be 6.5 in the classic group. Assuming a pH value of 7.0 in the Tulip group, with a standard deviation of 0.7, 64 patients are needed to achieve a power of 0.8 and an alpha value of 0.05 (PASS 15.0 software). An additional 10 % was added to account for potential dropouts, resulting in a total sample size of 72 participants. The assumed pH difference of 0.5 between groups was chosen based on the available literature [7] and served as the minimum detectable difference for the purpose of sample size estimation. This threshold has not been validated as a clinically meaningful cutoff, and our study was not powered to detect differences in clinical events such as regurgitation or aspiration.

Analysis of the primary outcome was performed in the modified intention-to-treat (mITT) population, defined as patients who received the study intervention and had the primary outcome measured. In our study, the per-protocol (PP) set was identical to the mITT set. Continuous data are presented as means with SDs or medians with interquartile range (IQR), depending on distribution. Continuous variables were analyzed using an independent *t*-test or rank sum test and the differences between groups were presented as the absolute mean difference (MD) and 95 % confidence interval (CI). Binary data are presented as number (%) and compared using the χ^2^ test or Fisher’s exact test, as appropriate. The differences between groups are presented as odds ratio (OR) with 95 % CIs. As less than 5 % of data were missing, handling of missing data was not applied. Prespecified subgroup analyses were performed according to BMI, sex, surgical site (abdominal vs. non-abdominal), surgical position, laparoscopic approach, and duration of anaesthesia. Subgroup analyses were not powered to detect interaction effects and should be considered exploratory. Sensitivity analysis included analysis of the primary outcome after excluding patients who were in head-down position during surgery.

We did not impute data for dropouts caused by surgery cancellations or protocol deviation after randomization. p<0.05 was considered statistically significant. All statistical analyses were performed using SPSS, version 26 (IBM, Armonk, NY, USA) and R 4.3.3 software (The R Project for Statistical Computing, www.r-project.org).

## Results

### Study population

From August 2024 to January 2025, a total of 129 patients were screened. Among them 57 patients were excluded by meeting exclusion criteria. A total of 72 patients were randomised, 36 to each group. One patient in the Tulip group had surgery cancelled. Finally, 71 patients finished the study and were involved in the mITT analysis (Figure 2). Baseline characteristics were comparable between the two groups (Table 1).

**Figure 2:**
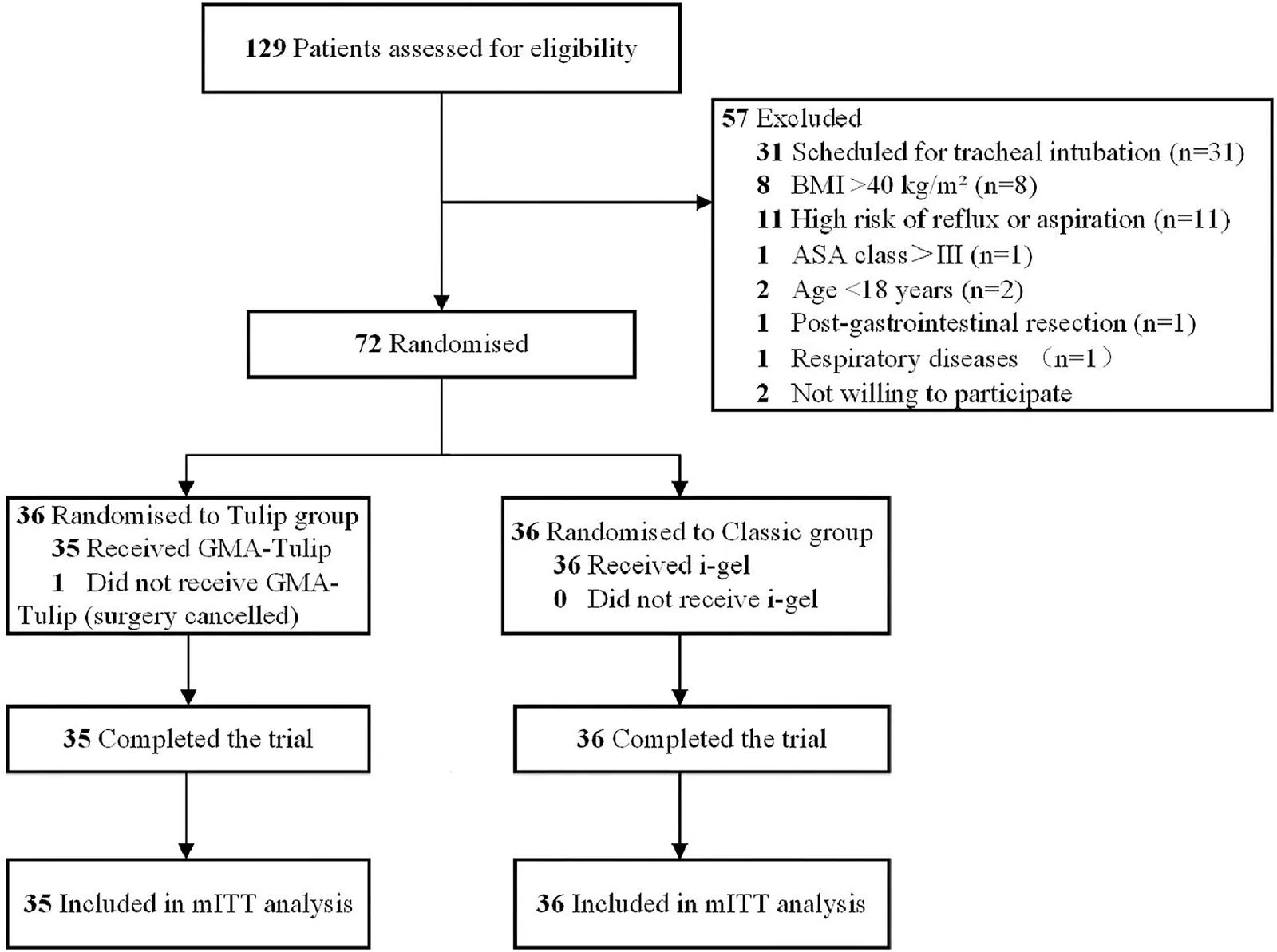
Flow chart of the study.

**Table 1:** Baseline characteristics.

Characteristics	Tulip (n=36)	Classic (n=36)
Age, yrs	52.3 ± 15.7	45.0 ± 13.6
Gender		
Male	14 (38.9)	14 (38.9)
Female	22 (61.1)	22 (61.1)
BMI, kg/m^2^	24.2 ± 2.6	23.9 ± 3.3
ASA status		
Ⅰ	4 (11.1)	5 (13.9)
Ⅱ	32 (88.9)	31 (86.1)
Location of lesion		
Gall bladder	8 (22.2)	10 (27.8)
Breast	12 (33.3)	13 (36.1)
Hernia	10 (27.8)	5 (13.9)
Others	6 (16.7)	8 (22.2)
Laparoscopic surgery		
Yes	14 (38.9)	15 (41.7)
No	22 (61.1)	21 (58.3)
Position during surgery		
Supine	20 (55.6)	22 (61.1)
Head-up	8 (22.2)	10 (27.8)
Head-down	8 (22.2)	4 (11.1)
Duration of surgery, min	77.8 ± 35.9	85.2 ± 43.4
Duration of anaesthesia, min	108.9 ± 39.7	115.7 ± 45.4

Data are presented as mean ± SD or frequency (%). BMI, body mass index; ASA, American society of anaesthesiologists.

### Primary and secondary endpoints

The results for the primary and secondary endpoints are summarised in Table 2. For the primary endpoint, the pH of secretion at the tip was comparable between the Tulip group (6.8 [0.7]) and the Classic group (6.9 [0.7]), with a MD of −0.04 (95 % CI: −0.37 to 0.30; p=0.830). No gastrooesophageal reflux was observed in either group. Moreover, the pH gradient between the tip and the back of the SAD was similar between groups (Tulip: 0.23 [0.23]; Classic: 0.29 [0.20]; p=0.620).

**Table 2:** Primary and secondary endpoints.

Endpoints	Tulip (n=35)	Classic (n=36)	MD or OR (95 % CI)	p-Value
Primary endpoint				
pH of secretion at tip, mean (SD)	6.8 (0.7)	6.9 (0.7)	−0.04 (−0.37, 0.30)	0.830
Secondary endpoint				
During induction				
OLP, cmH_2_O, mean (SD)	27.3 (8.5)	27.9 (8.5)	−0.60 (−4.64, 3.43)	0.766
Number of attempts, median (IQR)	1 (0)	1 (0.75)	0 (0, 0)	0.026
Time to insertion, sec, mean (SD)	15.8 (10.6)	26.3 (48.4)	−10.5 (−27.2, 6.3)	0.214
Time to effective ventilation, sec, mean (SD)	23.0 (10.7)	49.2 (64.8)	−26.2 (−48.3, −4.0)	0.022
Epiglottic downfolding, n (%)	5 (14.3)	11 (34.4)	0.32 (0.10, 1.05)	0.054
Fibroscopic grading of position, median (IQR)	0 (0)	0 (0)	0 (0, 0)	0.529
Difficulty of gastric tube insertion, median (IQR)insertion	0 (0)	0 (0)	0 (0, 0)	0.282
Intraoperative adjustments, n (%)	3 (8.6)	2 (5.6)	1.6 (0.3, 10.2)	0.674
End of surgery				
Gastroesophageal reflux, n (%)	0 (0)	0 (0)	/	1
pH gradient between tip and back of laryngeal mask, mean (SD)	0.23 ± 0.23	0.29 ± 0.20	−0.05 (−0.28, 0.17)	0.620
Blood staining, n (%)	2 (5.7)	0 (0)	/	0.239
Anaesthesiologist satisfaction with the SAD, median (IQR)	9 (1)	9 (1)	0 (0, 0)	0.885
PACU				
Sore throat, n (%)	12 (34.3)	6 (16.7)	2.61 (0.85, 8.00)	0.088
Hoarseness, n (%)	1 (2.9)	1 (2.8)	1.03 (0.06, 17.13)	0.984
Dysphonia, n (%)	1 (2.9)	0 (0)	/	0.493
PONV, n (%)	7 (20.0)	8 (22.2)	0.88 (0.28, 2.74)	0.819
24 h post-op				
Sore throat, n (%)	8 (22.9)	6 (16.7)	1.48 (0.46, 4.82)	0.512
Hoarseness, n (%)	1 (2.9)	0 (0)	/	0.493
Dysphonia, n (%)	0 (0)	0 (0)	/	1
PONV, n (%)	6 (17.1)	3 (8.3)	2.28 (0.52, 9.93)	0.265

Data are presented as mean ± SD, median (IQR) or number (%). SD, standard deviation; IQR, interquartile range; OLP, oropharyngeal leak pressure; PACU, post-anaesthesia care unit; PONV, postoperative nausea and vomiting; MD, mean difference; OR, odds ratio; CI, confidence interval.

The number of attempts was significantly lower in the Tulip group (median [Q1, Q3]: 1 [0]) compared to the Classic group (1 [0.75], p=0.026). Time to insertion did not differ significantly between groups (Tulip: 15.8 ± 10.6 s; Classic: 26.3 ± 48.4 s, p=0.214). However, the time to effective ventilation was significantly shorter in the Tulip group than in the Classic group (23.0 [10.7] vs. 49.2 [64.8] sec, MD: −26.2; 95 % CI: −48.3 to −4.0; p=0.022). OLP was similar between groups (27.3 [8.5] vs. 27.9 [8.5] cmH_2_O; p=0.766). The incidence of epiglottic downfolding was 14.3 % in the Tulip group and 34.4 % in the Classic group (p=0.054). Fibroscopic grading of position and difficulty of gastric tube insertion showed no significant differences. Intraoperative adjustments were required in 8.6 % of Tulip cases and 5.6 % of Classic cases (p=0.674). Upon removal, blood staining on the SAD was observed in 5.7 % of the Tulip group and none in the Classic group (p=0.239). Anaesthesiologist satisfaction scores did not differ between groups (9 [1] vs. 9 [1], p=0.885).

The incidence of sore throat in the PACU was 34.3 % in the Tulip group and 16.7 % in the Classic group (OR: 2.61; 95 % CI: 0.85–8.00; p=0.088). Incidences of hoarseness (2.9 % vs. 2.8 %), dysphonia (2.9 % vs. 0 %) and PONV (20.0 % vs. 22.2 %) in Tulip group did not differ between groups (p=0.984, 0.493 and 0.819, respectively). At 24 h postoperatively, the incidences of sore throat, hoarseness, dysphonia and PONV were not significantly different between the two groups.

### Subgroup analysis and sensitivity analysis

Subgroup analyses demonstrated no significant interaction effects between treatment group and age, sex, laparoscopic approach, surgical position, or anaesthesia duration (Figure 3). After excluding patients who underwent surgery in the Trendelenburg position, the primary outcome remained non-significant between groups (Tulip vs. Classic: 7.00 [0.61] vs. 6.83 [0.65], p=0.320). Post-hoc analysis showed that the first-attempt success rate was 94.3 % (33/35) in the Tulip group and 75.0 % (27/36) in the Classic group. No SAD placement failed or required conversion to tracheal intubation in either group.

**Figure 3:**
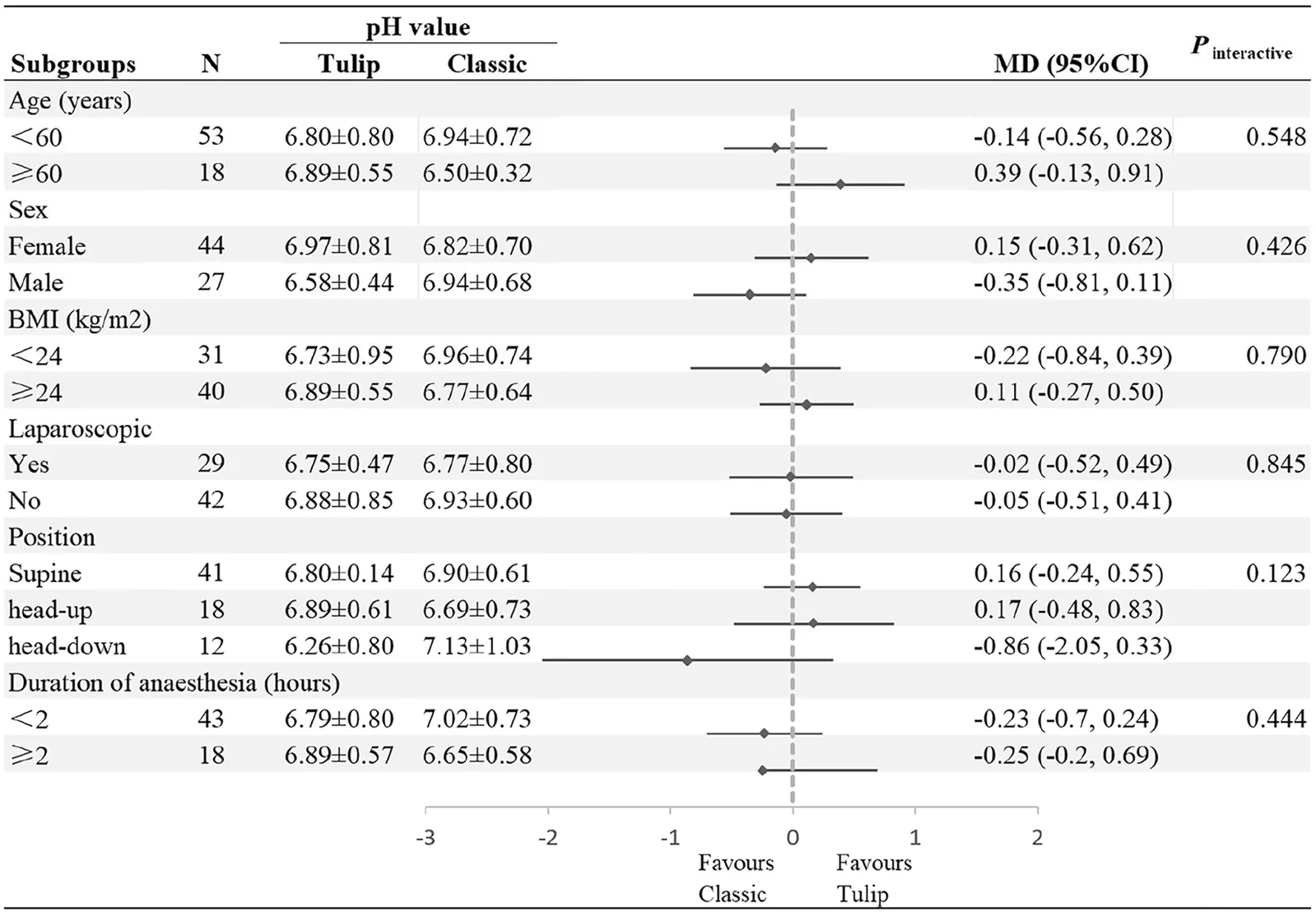
Forest plot of sub-group analysis.

## Discussion

In this randomised clinical trial, GMA-Tulip did not demonstrate a significant difference in the pH of secretions, an indirect measure of regurgitation, compared to i-gel. The theoretical mechanism by which GMA-Tulip might reduce regurgitation through sealing of the piriform fossae lacks supporting clinical evidence.

To our knowledge, this is the first randomised trial to evaluate the influence of tulip-shaped SAD design on regurgitation-related outcomes. In this study, we measured the pH value of secretions at the tip of the SAD, which reflects the pH in the hypopharynx and the ooesophageal inlet. Most previous studies on gastrooesophageal reflux during SAD ventilation have used pH probes to measure ooesophageal pH as an indicator of gastric content reflux. Some studies have measure pH at multipleooesophageal sites [8], or in both the oesophagus and hypopharynx [9]. Other studies have evaluated regurgitation by administering a methylene blue capsule before induction and inspecting the SAD and the oropharynx for bluish discoloration at the end of surgery [10]. Our method was adapted from Lemos’s study [7], as it is simpler and more convenient. The pH values we obtained (6.8 and 6.9 in the two groups, respectively) were similar to those reported by Lemos et al. (6.5) [7]. However, in Hagberg’s study measuring the pH value with probes, the pH value at hypopharynx was much lower (5.3) [9]. This discrepancy may be attributable to differences in measurement methods and possibly to the different types of SADs studied. Our study focused on gel SADs, while Hagberg’s study investigated LMA. A human cadaver pilot study reported that i-gel had lower incidence of regurgitation than LMA (40 % vs. 50 %) [11]. However, whether gel-based SADs confer a lower risk of reflux compared with LMAs needs more evidence. Furthermore, one study inserted pH probes at 30 cm and 20 cm from the anterior nares and found that only the pH value at 30 cm differed between LMA and endotracheal intubation [8]. Given that reflux reaching the hypopharynx is of greater clinical concern, we considered hypopharyngeal pH measurement a more relevant indicator of clinically significant reflux.

Mean time to insertion in the Classic group was 26.3 s, consistent with previously reported values for the i-gel (ranging from 21 s to 27 s) [12], [13], [14]. Although the difference in insertion time (15.8 s in the Tulip group) was not statistically significant, the shorter time to effective ventilation (by 10.5 s) suggests a potential advantage for GMA-Tulip. Furthermore, fewer insertion attempts were required in the Tulip group, though the clinical significance of this difference warrants further verification. However, operator-dependent secondary outcomes including insertion attempts, time to effective ventilation, intraoperative adjustments, and satisfaction scores were assessed by the unblinded proceduralist and are therefore susceptible to performance bias. These operator-dependent findings should be interpreted with appropriate caution.

The mean OLP observed in our study was 27.3 cmH_2_O in the Tulip group and 27.9 cmH_2_O in the Classic groups. It was recommended that the OLP should exceed 20 cmH_2_O, particularly during positive-pressure ventilation [15]. During laparoscopic surgery, when airway pressures are elevated, an OLP >25 cmH_2_O has been recommended, or at least 8 cmH_2_O above the peak airway pressure measured during normoventilation in the supine position. This may help to minimise the risk of air leaks and prevent the opening of the ooesophageal sphincter [6]. Thus, the OLP values observed in our study are within the recommended range. Furthermore, the OLP values of GMA-Tulip in our study are consistent with those reported by Zhang et al., who observed an OLP of 28.5 (1.5) cmH_2_O in the supine position [4].

Our results showed that 34.3 % of the patients in the Tulip group had sore throat, while 16.7 % in the Classic group, without significant difference. The incidence of sore throat for SADs varies markedly between trials and could be as high as 49 % [16]. However, the incidences of sore throat for i-gel are relatively low, varies between 2.9 % and 21.4 % [16], [17], [18]. Our results is in accordance with these reports. Although the differences in sore throat and blood staining between groups did not reach statistical significance, the numerically higher rates in the Tulip group deserve comment. The GMA-Tulip has a wider, tulip-shaped cuff designed to conform to the bilateral piriform fossae, which may theoretically improve anatomical fit but could also exert greater pressure on the pharyngeal mucosa, potentially increasing the risk of postoperative throat discomfort. Similarly, its more pronounced tip geometry might increase mucosal friction during insertion, as suggested by the slightly higher blood staining rate. However, these are secondary, operator-dependent outcomes, and the lack of statistical significance along with the absence of blinding of the proceduralist precludes definitive conclusions. These observations should be considered exploratory and require confirmation in future studies designed to assess airway-related morbidity.

Our study population was heterogeneous with respect to surgical type, laparoscopic approach, and patient positioning. While we performed subgroup and sensitivity analyses to assess the consistency of the primary outcome across these factors (Figure 3), we did not systematically record pneumoperitoneum pressure or airway pressures on a per-patient basis. However, all patients were managed under standardized institutional protocols, with pneumoperitoneum pressure maintained at 12–14 mmHg and peak airway pressures below 25 cmH_2_O. Future studies with more standardized protocols and comprehensive data collection would be valuable to further evaluate the influence of these variables on SAD-related regurgitation risk.

### Limitation

The main limitation of this trial is that the pH of the secretion at the tip of the laryngeal mask airway, used as an indicator of regurgitation, is a relatively indirect measure. We also measured the difference in pH between secretions at the back and the tip of the SAD. Theoretically, regurgitation would result in a lower pH at the tip than at the back. The results showed no significant difference in this pH gradient between the two groups, supporting the conclusion that there is no difference in regurgitation between the two types of SADs. Second, we did not restrict surgical positioning, so both head-down and head-up positions were included. The head-down position may increase the risk of regurgitation. However, a sensitivity analysis excluding patients in the head-down position yielded results consistent with the primary mITT analysis. Third, the sample size was calculated based on the primary outcome of secretion pH, a surrogate marker, and was not powered to detect clinically relevant events such as regurgitation or aspiration. Therefore, the absence of observed reflux in either group should not be interpreted as evidence of comparable safety between the two devices. Future studies with larger sample sizes and direct measures of regurgitation are warranted. Fourth, pH measurements were performed only once due to the limited volume of secretions, and the resolution of the pH strips (0.5 units) precludes detection of very small differences. These methodological factors should be considered when interpreting the primary outcome. Fifth, the attending anaesthesiologist performing SAD insertion could not be blinded due to the visibly different device designs. To minimize bias, the outcome assessors were independent and blinded to group allocation. Nevertheless, the lack of blinding of the proceduralist should be considered when interpreting the results.

## Conclusions

In this randomised trial, GMA-Tulip did not show a difference in the pH of hypopharyngeal secretions compared to i-gel. Whether this translates to a difference in clinically significant regurgitation or aspiration requires further investigation using direct measurement methods.
